# Cancer-associated fibroblast related gene signature in Helicobacter pylori-based subtypes of gastric carcinoma for prognosis and tumor microenvironment estimation *in silico* analysis

**DOI:** 10.3389/fmed.2023.1079470

**Published:** 2023-01-18

**Authors:** Ruofan Xu, Le Yang, Zhewen Zhang, Yuxuan Liao, Yao Yu, Dawei Zhou, Jiahao Li, Haoyu Guan, Wei Xiao

**Affiliations:** ^1^Department of Infectious Disease, Third Xiangya Hospital, Central South University, Changsha, Hunan, China; ^2^Xiangya School of Medicine, Central South University, Changsha, Hunan, China; ^3^National Cancer Center, National Clinical Research Center for Cancer, Cancer Hospital, Chinese Academy of Medical Sciences and Peking Union Medical College, Beijing, China; ^4^Graduate School of Peking Union Medical College, Chinese Academy of Medical Sciences and Peking Union Medical College, Beijing, China

**Keywords:** Helicobacter pylori, gastric cancer, cancer-associated fibroblasts, prognosis, tumor microenvironment

## Abstract

**Introduction:**

Gastric cancer (GC) remains the major constituent of cancer-related deaths and a global public health challenge with a high incidence rate. Helicobacter pylori (HP) plays an essential role in promoting the occurrence and progression of GC. Cancer-associated fibroblasts (CAFs) are regarded as a significant component in the tumor microenvironment (TME), which is related to the metastasis of GC. However, the regulation mechanisms of CAFs in HP-related GC are not elucidated thoroughly.

**Methods:**

HP-related genes (HRGs) were downloaded from the GSE84437 and TCGA-GC databases. The two databases were combined into one cohort for training. Furthermore, the consensus unsupervised clustering analysis was obtained to sort the training cohort into different groups for the identification of differential expression genes (DEGs). Weighted correlation network analysis (WGCNA) was performed to verify the correlation between the DEGs and cancer-associated fibroblasts which were key components in the tumor microenvironment. The least absolute shrinkage and selection operator (LASSO) was executed to find cancer-associated fibroblast-related differential expression genes (CDEGs) for the further establishment of a prognostic model.

**Results and discussion:**

In this study, 52 HP-related genes (HRGs) were screened out based on the GSE84437 and TCGA-GC databases. A total of 804 GC samples were analyzed, respectively, and clustered into two HP-related subtypes. The DEGs identified from the two subtypes were proved to have a relationship with TME. After WGCNA and LASSO, the CAFs-related module was identified, from which 21 gene signatures were confirmed. Then, a CDEGs-Score was constructed and its prediction efficiency in GC patients was conducted for validation. Overall, a highly precise nomogram was established for enhancing the adaptability of the CDEGs-Score. Furthermore, our findings revealed the applicability of CDEGs-Score in the sensitivity of chemotherapeutic drugs. In general, our research provided brand-new possibilities for comprehending HP-related GC, evaluating survival, and more efficient therapeutic strategies.

## Introduction

Gastric cancer (GC) is the third leading cause of cancer-related deaths worldwide (1), especially in East Asia. Worldwide, the number of newly diagnosed GC patients is about 990,000 per year. The mortality from GC is high with 784,000 deaths globally in 2018 (2). The incidence of GC varies considerably between genders and regions, specifically, the incidence rate in men is two to three times higher than that in women (3). The clinically available therapies for GC are quite restricted, and the median overall survival for advanced-stage gastric cancer is merely around 8 months (4). Notably, more than two decades of research have demonstrated an inextricable link between Helicobacter pylori (HP) and GC. HP is a Gram-negative bacillus, which is microaerobic and spiral in shape. It is usually found in stomach and is resistant to gastric acid. The evolution of its spiral shape enables it to penetrate the mucus lining. HP can cause a variety of gastrointestinal disorders, including GC. It is estimated that GC induced by HP accounts for 65–80% of all GC cases. The traditional view is that there are two potential pathways of HP pathogenesis in academic circles: inflammation-mediated damage to gastric epithelial cells and direct action of bacteria, respectively. On the other hand, HP can also interfere directly with the metabolism of epithelial cells by producing the bacterial agent cytotoxin-associated gene A (5). Despite the fact that comprehensive gastric cancer treatment can somewhat deter the aforementioned pathogenic mechanisms, the problem of tumor recurrence and metastasis faced by GC patients has remained unresolved. As research progresses, the limitations of GC treatment strategies are increasingly attributed to alterations in the tumor microenvironment (TME) mediated by tumor stromal activity, in which cancer-associated fibroblasts (CAFs) occupy an important position (6). In spite of this, the biological mechanism of HP on CAFs in GC remains unsolved.

More and more researches have witnessed that cell interactions play an overarching role in TME, which is associated with tumor metastasis (7). In detail, CAFs were associated to the differentiation of protumorigenic macrophage (8), suppression of NK cells (9), and blocking of the maturation of dendritic cells (10) *via* regulatory molecules in TME. Obviously, CAFs are an important component of the TME and were found in nearly every kind of solid tumor. CAFs have been verified to promote cancer growth by supporting tumor progression, remodeling the extracellular matrix, brokering tumor-related inflammation and facilitating angiogenesis (11). It is well known that normal fibroblasts already have an inhibitory role on cell proliferation and tumor cell motility *in vitro* (12), as that on epithelial carcinogenesis (13). Under this circumstance, normal CAFs are reprogrammed into tumorigenic ones. The aforementioned transformation requires a vast number of cancerogenic causes and particular TME motivation, such as oxidative stress and hypoxia, by which recruitment and activation of fibroblasts are enhanced. Within these, TGFβ7 (14), PDGF62 (15), and IL-6 (16) are known fibroblast activators and participate in cellular signaling circuits with their corresponding receptors, thus mediating their enhancement of synthesis and secretion capabilities. Not coincidentally, these general findings are also confirmed in the TME of HP-associated GC. By harvesting stomach samples from 8-week-old Spraque–Dowley rats for incubation with the HP strain, the researchers found that in the long-term presence of exosomes secreted by HP-activated gastric fibroblasts (HP-AGF), normal gastric epithelial cells undergo a cancer stem cell-associated programmed transformation, and this type of transformation is associated with cancer development and metastasis (17). In particular, HP-AGF further promotes the reprogramming of normal cells to a tumor-like phenotype by inducing the aggregation of actin to the nucleus and interfering with the process of DNA transcription and repair in cells (18, 19). Given that HP and CAFs play a key role in the formation of TME, it is interesting to explore how HP-associated GC has altered TME infiltration and exacerbated disease progression in GC patients with the help of CAFs.

In our study, we made a prospective analysis of the expression profiles of HP-related genes (HRGs). HP-associated-fibrosis relative-gastric cancer differential expression genes expression (DEGs) were also explored to validate the correlation with TME and prognosis of GC patients in Gene Expression Omnibus (GEO) and The Cancer Genome Atlas (TCGA) databases. And we further built a prognostic model based on different tumor subtypes with the help of DEGs. Moreover, the GC metastasis and recurrence faced by patients and medical staff are still a severe challenge (20). Recent years have witnessed the continuous revolution in the field of chemotherapy which has become a main medical strategy for advanced GC (21). Consequently, a comprehensive analysis of the mechanisms and characteristics of CAFs in the TME is able to point out new ways to reveal the crucial processes of carcinogenesis in GC and further carry out chemotherapy sensitivity prediction.

**FIGURE 1:**
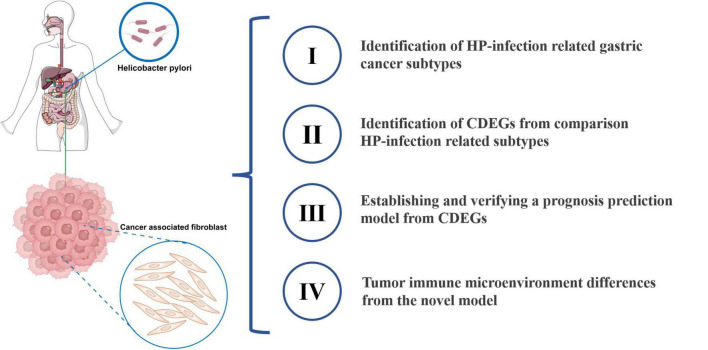
Diagram of the study. HP, Helicobacter pylori; CDEGs, cancer associated fibroblast related differential expression genes.

The whole process of this study was exhibited in Figure 1.

## Materials and methods

### Data sources and pretreatment

Clinicopathological data in GC samples were retrieved from GEO^1^ (GSE84437) and TCGA^2^ (TCGA-GC). All of the data was high-throughput gene expression (fragments per kilobase million, FPKM). We acquired the original “CELL” documents and obtained quantile normalization and background adjustment. The data of GSE84437 and TCGA-GC were implemented conversion from FPKM values to transcripts per kilobase million (TPM) using the following formula:


$$T\,P\,M_{i}=\frac{F\,P\,K\,M_{i}}{T\,o\,t\,a\,l\,l\,i\,b\,r\,a\,r\,y\,F\,P\,K\,M}\times 10^{6}$$

We then aggregated the two datasets for the sequent operations. The batch effects were eliminated after applying the “Combat” algorithm through “SVA” R package. Ultimately, there were 804 GC patients being included for further research after excluding data with no survival information. The clinical parameters contained sex, age, TNM stage, survival status, and follow-up time.

### HRGs cluster analysis

The HRGs were downloaded in MSigDB.^3^ The full details of these genes were shown in Supplementary Table 1. To evaluate the prognostic value of HRGs, GSE84437 and TCGA were combined into one cohort for training. Univariate Cox regression analysis enumerated the whole HRGs and their corresponding *p*-value. HRGs with a *p*-value < 0.05 were regarded as prognostic-related genes. Furthermore, the consensus unsupervised clustering analysis was obtained to sort the training cohort into different groups based on the expression of prognostic-related genes with help of the “ConsensusClusterPlus” package. The HRGs cluster analysis was based on the following assumptions: Primarily, the cumulative distribution function (CDF) curve should meet continuity and stability. Next, the sample size was enough. Finally, the inter-group relation declined and the intra-group relation increased after clustering.

### Correlation between two subtypes with the clinical characteristics, immune infiltration, and related pathways in GC

After consensus clustering, we explored the association between the two subtypes. The patient features contained age, gender, TNM stage, and project. Further, the distinctions in immune infiltration among the two subtypes were observed executing the calibration algorithm. Moreover, we used Kaplan–Meier curves to identify the variance in recurrence-free survival (RFS) among different subtypes with the help of “survminer” and “survival” packages. The “GSVA” R package was performed to conduct gene set variation analysis (GSVA), which revealed the biological processes in different subtypes.

### DEGs identification in subtypes and functional annotation

Differential expression genes (DEGs) between the two subtypes were ascertained by the “limma” package according to a prerequisite that the modified *p*-value < 0.05 and the | Log_2_Fold Change| > 1. Meanwhile, principal component analysis (PCA) was performed with the “ggplot2” package. In order to demonstrate the function of the DEGs and explore the enriched pathways and associated gene functions, the Gene Ontology (GO) and Kyoto Encyclopedia of Genes and Genomes (KEGG) analyses were performed by the “clusterprofiler” R package.

### Module related with CAFs identification *via* WGCNA

After the function enrichment of DEGs in two subtypes, we hoped to investigate the correlation between the regulatory process of these DEGs intervened by HP and TME. To estimate the proportion of major cells in TME, Estimated the Proportion of Immune and Cancer Cells (EPIC) was used for the assessment of TME component with the help of “EPIC” R package. Furthermore, weighted correlation network analysis (WGCNA) was carried out to construct a DEGs-related gene cop-expressed network with “WGCNA” package. Concrete criteria were as followed: First, the gene expression level upload procedure was performed by R language. The network connection was calculated with help of Pearson’s correlation coefficient. Then, set the soft thresholding β as 5 to render the mean connectivity desirable among genes. Further, the transformation from the adjacency matrix to topological overlap matrix (TOM) was executed for subsequent gene hierarchical cluster by “hclust” algorithm. Finally, modules were identified with the utilization of dynamic tree cut and hierarchical cluster. Phenotypes and eigengenes network were used to assess the correlation between module and trait. The relationship between different cells and module eigengene was regarded as the definition of module membership. Module turquoise had the highest correlation coefficient to CAFs and was chosen for further study.

### Construction and validation of the CDEGs prognostic risk score

Cancer-associated fibroblasts (CAFs) related module was applied to the “survival” R package for univariate Cox regression on the basis of that *p < 0.05 was considered as the cut-off value. Then, performing the R package “glmnet,” these genes were included in least absolute shrinkage and selection operator (LASSO) regression to avoid overfitting. After that, 21 genes were screened out through the multivariate Cox analysis and regarded as cancer associated fibroblast related differential expression genes (CDEGs). The calculation of risk score was as follows: CDEGs-Score = sum of each gene’s (gene expression value × regression coefficient). GC patients were segregated into high- or low-risk groups according to the median risk score value. Later, Kaplan–Meier analysis and Log rank test were used to carry out the survival analysis of the two groups. Meanwhile, all groups underwent receiver operating characteristic (ROC) curves analysis.

### Establishment and verification of a nomogram scoring system

After independent prognosis analysis, the “rms” R package was used to produce a nomogram on the basis of the risk score clinical characteristics. Each parameter was associated with a given score respectively in the nomogram. The overall score obtained by summing the scores of each parameter was the sample score. In this nomogram, the actual observed data and the 1-, 3-, and 5-year survivals were presented on calibration plots.

### Combined characteristics analysis of the risk score

The “mafTools” R package was executed to obtain the mutation annotation format so as to explore the somatic mutations of GC patients in different groups. We analyzed the relation between 22 infiltrating immune cells and 21 prognostic CDEGs. In the high- and low-risk groups, we then applied boxplots to figure out the differential expression levels and cancer stem cell index. For each patient, the TME score was also calculated. In order to explore the association between the sensitivity to chemotherapeutic drugs and CDEGs-Score, the semi-inhibitory concentration (IC50) values were calculated to estimate clinically common drugs separately through “pRRophetic” R package.

### Statistical analysis

Statistical processing and analyses were executed using R software^4^ (version 4.1.0). All data were analyzed with significance at *p < 0.05.

## Results

### The landscape of HRGs in GC

**FIGURE 2:**
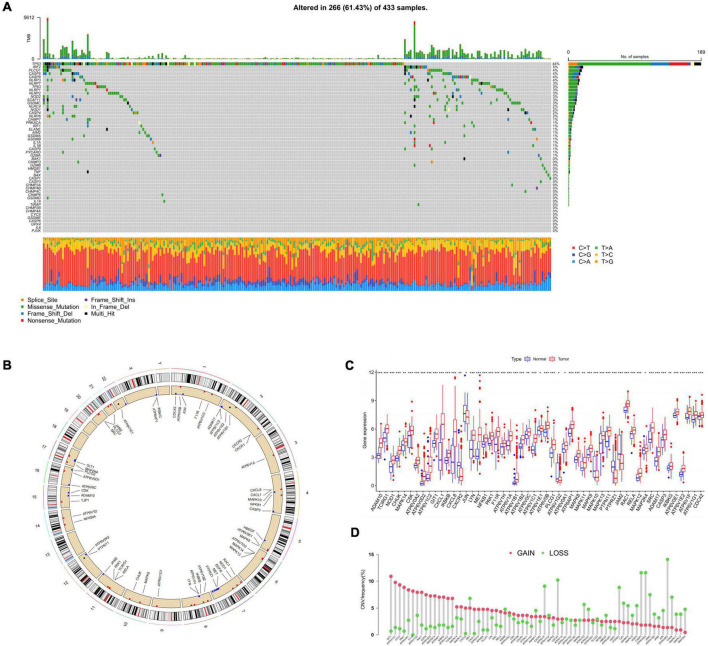
The landscape of HRGs in GC. **(A)** Frequencies of mutation in 433 patients with GC in GEO and TCGA datasets. The mutation frequency of each gene was presented on the right. Every column represented a sample. **(B)** CNV alterations of HRGs on 23 chromosomes. The red dot represented the gain frequency while the green dot represented the loss frequency. **(C)** Expression distributions of 52 HRGs between normal and GC samples, *p < 0.05, **p < 0.01, ***p < 0.001. **(D)** The CNV frequency of HRGs in all samples. HRGs, HP-related genes; GC, gastric cancer; GEO, Gene Expression Omnibus; TCGA, The Cancer Genome Atlas; CNV, copy number variations.

In two datasets, we screened out 52 HRGs and presented their mutation conditions with 433 HP-associated GC samples (Figure 2A), which showed a high mutation frequency (266, 61%). Among these genes, the one with the top mutation frequency was TP53 (44%). Following that were IRF2 (5%), PLCG1 (4%), CASP5 (4%), and CASP8 (4%). Among these alterations, missense mutations were the most common form, with T > A alterations predominating.

Next, somatic copy number variations (CNV) frequency analysis was performed on the HRGs with the boxplot. Figure 2B exhibited the position of CNV alterations in the HRGs on their relevant chromosomes. All 52 genes were observed to vary degrees of CNV in the mixed samples, among which GIT1, ATP6V1C1, CCL5, PAK1, and ATP6V0A1 had extensive CNV increases, while ATP6V0E2, CASP3, ATP6V0A4, and CDC42 presented CNV decreases (Figure 2D). We further investigated the mRNA levels in GC compared with normal samples and discovered that the mRNA level and CNV alteration showed no significant correlation in terms of HRGs (Figure 2C). There was a higher expression level of GIT1, ATP6V1C1, and PAK1 in GC samples than in normal samples. However, compared with the normal samples, HRGs such as CASP3 and CDC42 which were considered as CNV loss were also observed of higher expression level in GC ones. CXCR2, JUN, ATPV1G2, MAPK10, PTPRZ1, and JAM2 were the only HRGs lower expressed in GC samples, which didn’t show a consistent decrease in CNV alteration either. In general, it could be concluded that GC samples exhibited a generally high expression compared to normal tissues and this phenomenon was not exclusively related to CNV. Our findings demonstrated considerable differences in both gene profiles and HRG expression levels between different GC samples, suggesting a potential function of HRG in HP-related GC progression.

### Survival analysis of HRGs in HP-related GC patients

**FIGURE 3:**
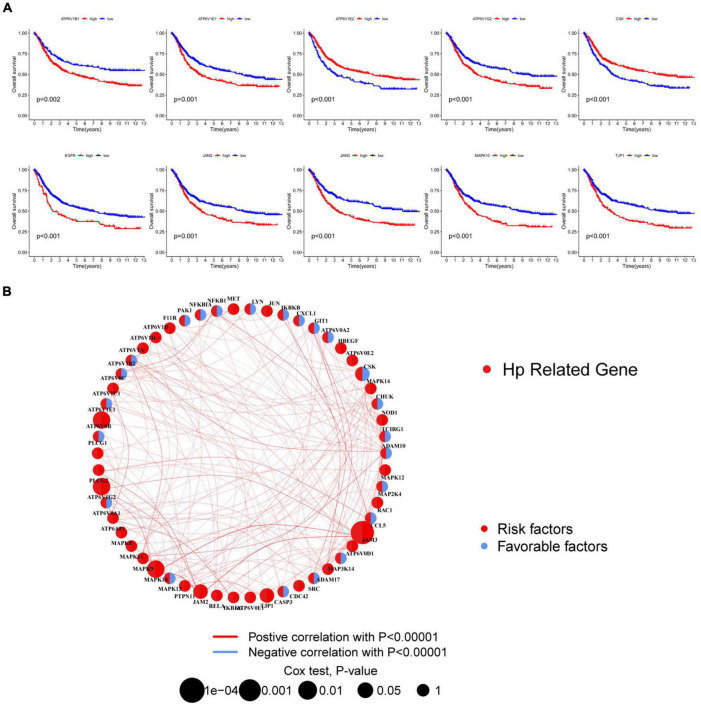
Survival analysis of HRGs in HP-related GC patients. **(A)** The Kaplan–Meier survival analysis. **(B)** Interactions among HRGs. The area of the circles represented the prognostic significance of each gene. The red circles, risk factors; the blue circles, protective factors. The correlation of genes was indicated by lines whose thickness presented the strength of connection. The red lines, positive associations; the blue lines, negative associations. HRGs, HP-related genes; GC, gastric cancer.

To further demonstrate the prognostic value of HRGs in GC, there were 9 genes being regarded as outstandingly prognostic genes in overall survival through analysis of univariate Cox regression and Kaplan–Meier survival (Figure 3A). Notably, ATP6V1B1 had relatively high prognostic significance (log-rank test, *p = 0.002). The combined landscape of HRG connection, interactions of the regulator, and prognostic value was illustrated in Figure 3B.

### Identification of HP-infection related subtypes and TME analysis

**FIGURE 4:**
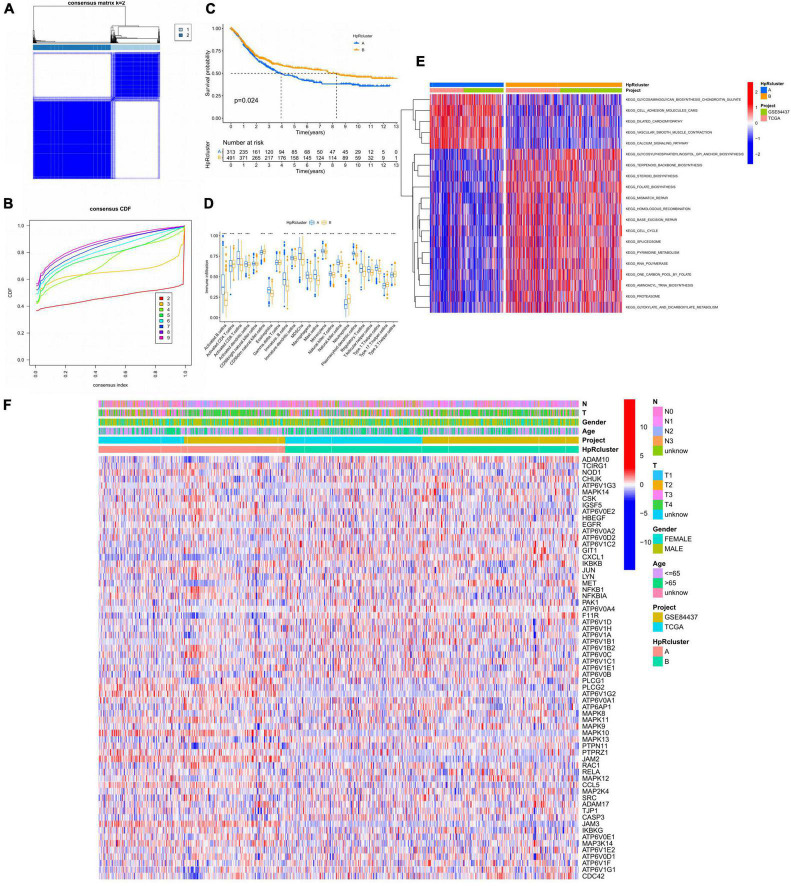
HP-related genes (HRGs) subtypes, clinicopathological and TME characteristics of subtype A and B classified by consensus clustering analysis. **(A)** Heatmap of consensus matrix (*k* = 2) showing the relative area of each cluster. **(B)** The CDF curves revealing the consensus distributions according to the *k* value. **(C)** Univariate analysis presenting 52 HRGs related to respective RFS time. **(D)** Immune infiltration level of 23 immune cells in subtype A and B, *p < 0.05, **p < 0.01, and ***p < 0.001. **(E)** GSVA between subtype A and B. Red represented activated pathways while blue represented suppressive pathways. **(F)** Distinct clinicopathological characteristics and expression levels of HRGs on account of different clusters. HRGs, HP-related genes; TME, tumor microenvironment; CDF, cumulative distribution function; GC, gastric cancer; RFS, recurrence-free survival; GSVA, gene set variation analysis.

To further figure out the expression characteristics of HRGs in GC, our study categorized GC patients by consensus clustering algorithm, which was according to the expression profiles of 52 HRGs. Our outcomes showed that *k* = 2 was optimum for separating the whole dataset into two subtypes (A, *n* = 313; B, *n* = 491; Figure 4A). On the other hand, by the measure of proportion of ambiguous clustering, when *k* = 2, the CDF curve slope was the smallest (Figure 4B). Next, the Kaplan–Meier curves obviously witnessed a better prognosis in subtype B on account of RFS with *p = 0.024 in log-rank test (Figure 4C).

Based on the clustering analysis, immune cell infiltration of TME was evaluated in the two subtypes, which displayed remarkable differences (Figure 4D). The activated B and CD8 T cells, eosinophils, immature B cells, macrophages, MDSCs, mast cells, regulatory T cells, follicular helper T cells, and type 1 helper cells had a significantly higher infiltration level in the subtype A, while only neutrophils exacted the opposite. Thus, immune cell infiltration analysis revealed a higher immune cells enrichment in subtype A, suggesting that immune factors might be a prognostic risk factor for HP-related GC. Meanwhile, the two subtypes were performed GSVA analysis and the subtype A showed an enrichment in extracellular matrix remodeling and cell adhesion related pathways based on two cohorts (GSE84437, TCGA) (Figure 4E). For instance, cell adhesion molecules, vascular smooth muscle, and dilated cardiomyopathy pathways were highly stimulated in subtype A. From another angle, the subtype B was significantly enriched in synthesis and metabolic pathways. In detail, it was manifested by extensive activation of terpenoids backbone, steroids, folate, glycosylphosphatidylinositol, spliceosome, RNA polymerase, and proteasome synthesis pathways, including pyrimidine, glyoxylate, and dicarboxylate metabolism pathways. In addition, gene damage repair pathways were also presented a highly activated state in subtype B, such as mismatch repair, homologous recombination, and base excision repair pathways.

Moreover, the expression of HRGs and clinicopathological features were relatively different in two subtypes (Figure 4F and Table 1). Subtype A was more inclined to present higher T stage and lower age (*p* < 0.001) compared to subtype B.

**TABLE 1:** The chi-square test of the relationship between two subtypes and clinical characteristics.

Characteristic	B	A	*p*
*n*	491	313	
Gender, *n* (%)			1.000
Female	165 (20.5%)	105 (13.1%)	
Male	326 (40.5%)	208 (25.9%)	
T, *n* (%)			<0.001
T1	27 (3.4%)	2 (0.2%)	
T2	82 (10.2%)	34 (4.2%)	
T3	155 (19.3%)	104 (12.9%)	
T4	221 (27.5%)	171 (21.3%)	
Unknown	6 (0.7%)	2 (0.2%)	
N, *n* (%)			0.560
N0	118 (14.7%)	70 (8.7%)	
N1	178 (22.1%)	107 (13.3%)	
N2	117 (14.6%)	89 (11.1%)	
N3	65 (8.1%)	42 (5.2%)	
Unknown	13 (1.6%)	5 (0.6%)	
Age, median (IQR)	65 (57, 72)	61 (53, 69)	<0.001

### Identification of DEGs and the corresponding functional enrichment

**FIGURE 5:**
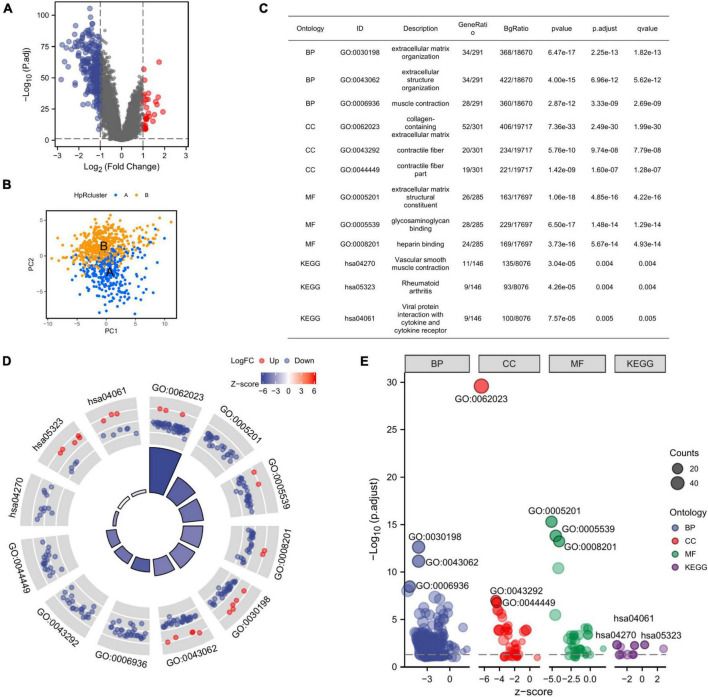
Identification of DEGs between the two subtypes and the corresponding functional enrichment. **(A)** A volcano plot corresponding to the regulation of DEGs in two subtypes, the default set of threshold was foldchange ≥ 2. **(B)** PCA plot exhibiting the distinctions in transcriptomes between the subtype A and B. **(C)** The description of GO terms and KEGG pathways of DEGs. **(D)** GO and KEGG analysis of DEGs (the top 12 are shown). **(E)** GO Bubble plot displaying expression level of DEGs in different terms. DEGs, differential expression genes; GO, Gene Ontology; KEGG, Kyoto Encyclopedia of Genes and Genomes; PCA, principal component analysis.

We further identified the DEGs in two subtypes and the discovery was displayed on the volcano plot (Figure 5A). Then, our PCA analysis indicated prominent difference between the two subtypes in the transcription profiles of HRGs (Figure 5B).

Given that there was a significant distinction between two subtypes clustered from HRGs, we further focused on what function the DEGs mainly played in subtype A and subtype B. Thus, we resorted to GO analysis for further confirmation. GO enrichment analyzed the DEGs associated with HRGs subtypes and the DEGs were found presenting an enrichment in GO terms correlated to extracellular matrix, which revealed the suppression of expression (Figure 5D). The extracellular matrix was significantly inhibited in organization, muscle contraction, collagen-containing, contractile fiber, and contractile fiber part indicating that the DEGs might have an internal relationship with the alteration of extracellular tissues and cells. Meanwhile, the molecular function enrichment results also displayed the inhibition of expression related to extracellular matrix (Figures 5C, E). The above results suggested that the DEGs had connections with extracellular matrix remodeling and cell adhesion, which implied that the DEGs potentially induced GC development by the alteration of TME. Considering the function of CAFs in TME, the DEGs was likely to influence the GC progression by the mediation of CAFs.

### Identification of CAFs-related module from comparing HP-infection related subtypes

**FIGURE 6:**
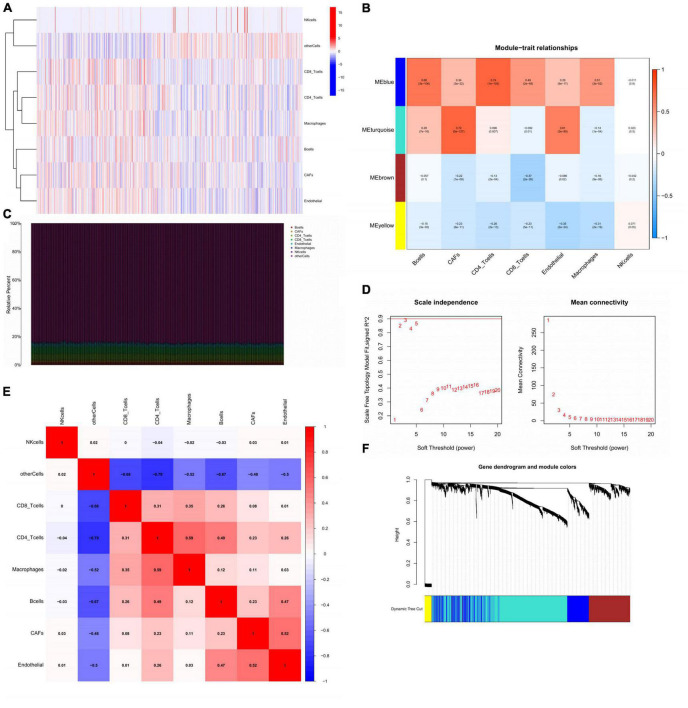
Identification of CAFs-related module from comparing HP-infection related subtypes. **(A)** The display of the expression status of different cells in GC. **(B)** Heatmap showing the relationship between GC traits and ME. Correlation coefficients and *P*-values were showed in each kind of cells. **(C)** Stacked bar plot showing the percentage of various cells, and cell fractions estimated by EPIC. **(D)** Identification of soft-threshold power through analyzing the value of mean connectivity and scale-free index. **(E)** The correlation matrix of eight types of GC sample cells. Coefficients were labeled. **(F)** Plot of gene dendrogram presenting the module assignment with the help of average linkage hierarchical clustering. CAFs, cancer associated fibroblasts; GC, gastric cancer; ME, module eigengenes; EPIC, estimating the proportions of immune and cancer cells.

The transcription level of each kind of cells in mixed samples sequency was showed (Figure 6A). Different cells had significant distinctions in expression profiles. Further, the proportion of B cells was the largest among all samples (>80%). The content of CAFs showed a rather stable share although the proportion was not high (Figure 6C). On this basis, we established a co-expression network based on the interactions between genes with the help of WGCNA. To render the topology network scale-free, soft threshold = 5 was considered as the optimal (Figure 6D). After clustering, 4 modules were identified by the hierarchical clustering tree (Figure 6F). The turquoise module was recognized as the CAFs-related module (Cor = 0.72, *p* = 5e-127) and was also highly related to endothelial (Cor = 0.61, *p* = 3e-80) (Figure 6B). Notably, the correlation of B cells with ME blue and ME turquoise was completely opposite to that of CAFs, suggesting that B cells might play an antagonistic role against CAFs during GC progression.

In addition, we explored the correlation between different cells by correlation analysis. In the correlation heat map, all immune cells except NK cells were correlated with each other in different degrees (Figure 6E). Importantly, CAFs showed a significant synergistic relationship with endothelial, suggesting that epithelial-mesenchymal transition might occur in TME under the action of CAFs.

### Establishing a prognosis prediction model from CDEGs

**FIGURE 7:**
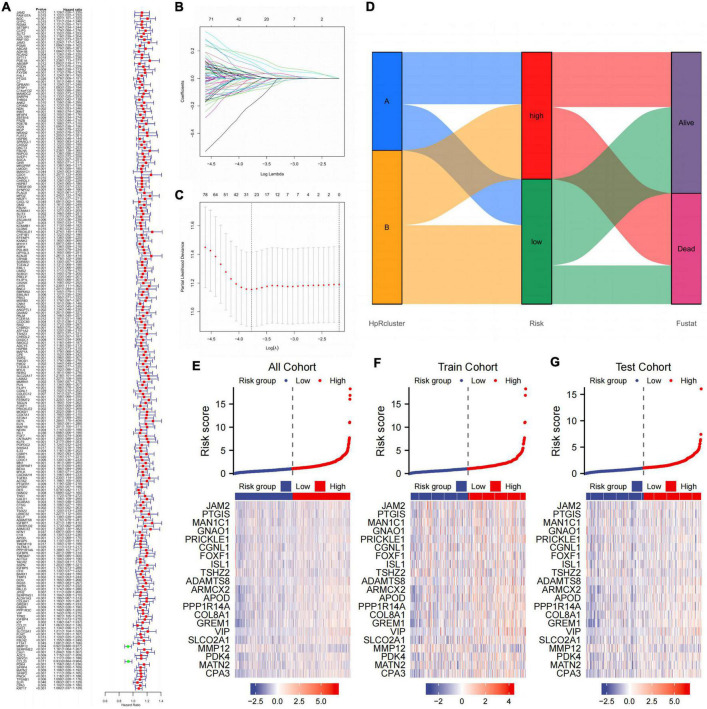
Establishing a prognosis prediction model from CDEGs. **(A)** Univariate COX regression confirmed that HRGs significantly related to the prognosis of GC. **(B,C)** LASSO and multivariate Cox regression analysis. **(D)** Alluvial diagram of subtype distributions in groups with different risk and fustat. **(E–G)** Heat map showing the correlation between gene expression and risk score in all cohorts, train cohort, and test cohort. CDEGs, cancer associated fibroblast related differential expression genes; HRGs, HP-related genes; GC, gastric cancer; LASSO, least absolute shrinkage and selection operator.

Before building the prediction model, we divided the cohort into two parts, the train cohort and the test cohort. The two cohorts were performed chi-square test to prevent confounding factors (Table 2). 228 genes were processed after preliminary analysis by univariate Cox regression. All included genes met the standard that *p < 0.05 and the hazard ratio range did not contain 1 (Figure 7A). Subsequently, a 21-gene signature was ascertained in accordance with the desirable l value with help of LASSO and multivariate Cox regression analysis (Figures 7B, C). Furthermore, we established the CDEGs-Score and its calculation formula was as follows:


$$C\,D\,E\,G\,s-S\,c\,o\,r\,e=\sum_{i=1}^{21}\beta_{n}\times {\left[F\,P\,K\,M\right]}_{n}$$

**TABLE 2:** The chi-square test of the relation between train set and test set.

Characteristic	Train	Test	*p*
*n*	402	402	
Gender, *n* (%)			1.000
Female	135 (16.8%)	135 (16.8%)	
Male	267 (33.2%)	267 (33.2%)	
T, *n* (%)			0.708
T1	11 (1.4%)	18 (2.2%)	
T2	56 (7%)	60 (7.5%)	
T3	134 (16.7%)	125 (15.5%)	
T4	197 (24.5%)	195 (24.3%)	
Unknown	4 (0.5%)	4 (0.5%)	
N, *n* (%)			0.505
N0	94 (11.7%)	94 (11.7%)	
N1	139 (17.3%)	146 (18.2%)	
N2	104 (12.9%)	102 (12.7%)	
N3	59 (7.3%)	48 (6%)	
Unknown	6 (0.7%)	12 (1.5%)	
Age, median (IQR)	64 (56, 71)	63 (55, 71)	0.332

The whole cohort was categorized into high- and low-score groups according to the median risk score. The samples numbers in each group were 405 and 399, respectively. In addition, comparison of the clinical materials between two risk groups revealed that the clinicopathological features were relatively different among two groups, which mainly showed in T (*p = 0.004) and N (*p = 0.002) stage (Table 3). After dividing the cohort into two groups, the alluvial presented a strong correlation between high-risk and dead outcome. For the majority of cases in the high-risk group had a survival outcome of death, while the majority of cases in the low-risk group survived instead. Nevertheless, there was no considerable discrepancy between risk and HP-related subtypes (Figure 7D).

**TABLE 3:** The chi-square test of the relation between two risk groups and clinical features in cohort.

Characteristic	Low	High	*p*
*N*	399	405	
Gender, *n* (%)			0.940
Female	135 (16.8%)	135 (16.8%)	
Male	264 (32.8%)	270 (33.6%)	
T, *n* (%)			0.004
T1	22 (2.7%)	7 (0.9%)	
T2	62 (7.7%)	54 (6.7%)	
T3	139 (17.3%)	120 (14.9%)	
T4	173 (21.5%)	219 (27.2%)	
Unknown	3 (0.4%)	5 (0.6%)	
N, *n* (%)			0.002
N0	117 (14.6%)	71 (8.8%)	
N1	135 (16.8%)	150 (18.7%)	
N2	87 (10.8%)	119 (14.8%)	
N3	52 (6.5%)	55 (6.8%)	
Unknown	8 (1%)	10 (1.2%)	
Age, median (IQR)	63 (54, 71)	64 (56, 71)	0.124

Further, we compared gene expression differences in three different cohorts with the risk factor maps (Figures 7E–G). It was evident that 21 genes showed expression differences between two group in all three cohorts.

### Validation of the CDEGs-related model

**FIGURE 8:**
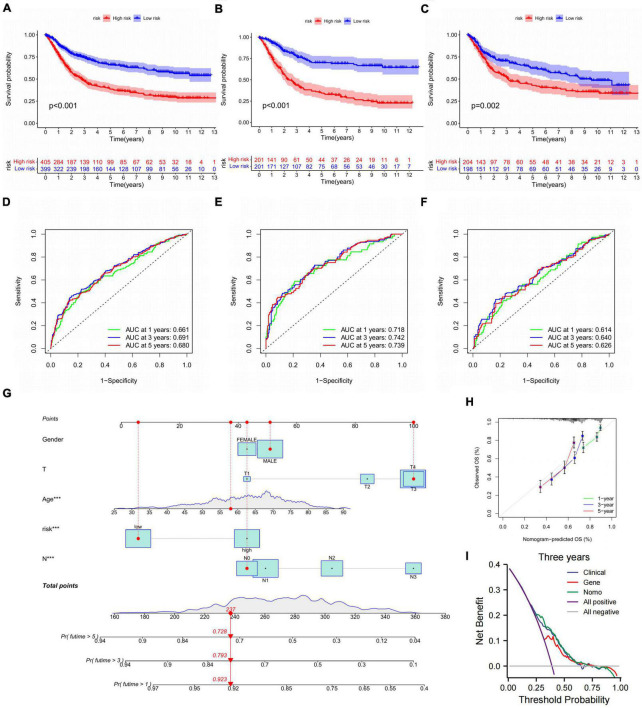
Validation of the CDEGs-related model. **(A–C)** The Kaplan–Meier survival analysis of all cohorts, train cohort, and test cohort. **(D–F)** ROC curves comparing the 1-, 3-, and 5-year overall survival in all cohort, train cohort, and train cohort. **(G)** Nomogram for predicting the 1-, 3-, and 5-year overall survival in the train cohort, ***p < 0.001. **(H)** Calibration curves of the nomogram for predicting of 1-, 3-, and 5-year overall survival. **(I)** DCA curves for two independent prognostic factors or a combination of them in OS prediction. CDEGs, cancer associated fibroblast related differential expression genes; ROC, receiver operating characteristic; DCA, decision curve analysis; OS, overall survival.

On account of the prognosis prediction model, the high- and low-risk groups were exhibited Kaplan–Meier survival analysis in three validations (Figures 8A–C). There was an apparent difference in survival probability between two groups in all cohorts (*p < 0.001), train cohort (*p < 0.001), and test cohort (*p = 0.002). On the basis of the risk score, the AUC values of 1-, 3-, and 5-year overall survival (OS) for all cohorts were 0.661, 0.691, and 0.680, respectively (Figure 8D). The AUC values of 1-, 3-, and 5-year OS for the train cohort predicted by the risk score were 0.718, 0.742, and 0.739, respectively (Figure 8E). The AUC values for the test cohort were 0.614, 0.640, and 0.626 at 1, 3, and 5 years in OS predicted by the risk score (Figure 8F). Given that the clinical application of CDEGs-Score in calculating OS was inconvenient in patients, the nomogram relying on the risk and clinical characteristics was constructed to predict the OS rates at 1, 3, and 5 years (Figure 8G). Calibration curves were applied to ensure that the nomogram displayed an excellent consistence between observation and prediction (Figure 8H). Moreover, the decision curve analysis (DCA) indicated that the combination prediction of CDEGs and clinical factor was superior to the TNM stage (Figure 8I).

### Exploring the factors influencing the prognosis of different groups

**FIGURE 9:**
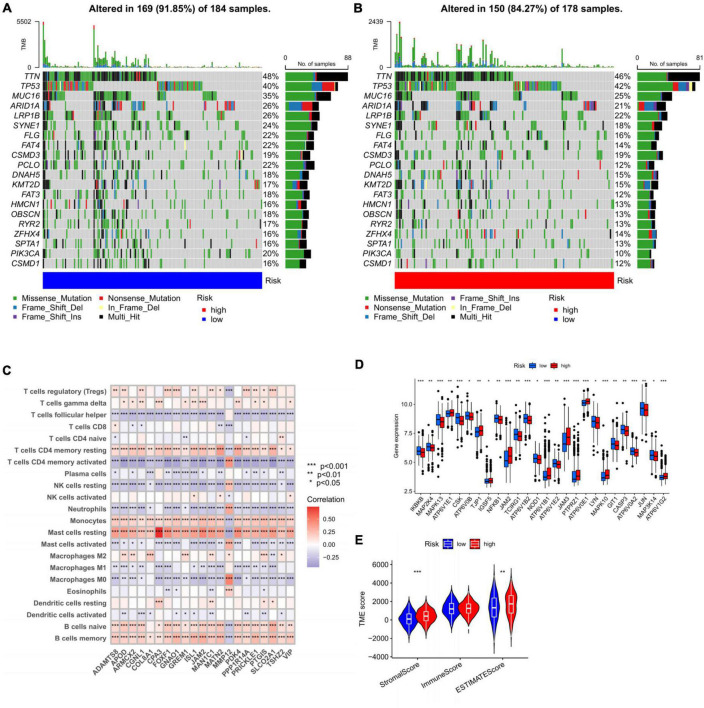
Exploring the factors influencing the prognosis of different groups. **(A,B)** The waterfall plot of somatic mutation features constructed with high- and low-risk groups. **(C)** Correlations between the abundance of immune cells and 21 genes in the prediction model, *p < 0.05, **p < 0.01, and ***p < 0.001. **(D)** Expression distributions between high- and low-risk groups in HGRs. **(E)** The correlations between risk and TME score. TME, tumor microenvironment.

Then the distribution variations of the somatic mutations were analyzed between high- and low-risk groups in all cohort. The top 20 mutated genes were *TTN*, *TP53*, *MUC16*, *ARID1A*, *LRP1B*, *SYNE1*, *FLG*, *FAT4*, *CSMD3*, *PCLO*, *DNAH5*, *KMT2D*, *FAT3*, *HMCN1*, *OBSCN*, *RYR2*, *ZFHX4*, *SPTA1*, *PIK3CA*, and *CSMD1* (Figures 9A, B). Compared to low-risk group, high-risk group gained higher frequencies of *TTN* and *TP53* mutations relatively. However, the decisive difference was identified due to the mutation levels of *MUC16*, *PCLO*, and *PIK3CA*. We next explored the connection between the 21 genes in the prognosis prediction model and the enrichment of immune cells. Our results found most immune cells were significantly related to these genes (Figure 9C). Monocytes, resting mast cells, naïve B cells, and memory B cells showed a significant positive correlation with 21 genes, which suggested that these cells were involved in disease progression and contributed to different prognosis in high- and low-risk groups, respectively. In addition, we assessed the expression distributions of the HRGs between the two groups. The boxplot presented that the expression level of 26 genes were obviously different, including TJP1, JAM2, ATP6V1E2, and JAM3 (Figure 9D).

Apart from this, we hoped to fix out the relationship of risk with TME score. For the TME score, higher stromal and estimate scores was associated with high-risk group, which demonstrated higher TME scores for patients in high-risk group(Figure 9E).

### Drug sensitivity analysis based on CDEGs-score

**FIGURE 10:**
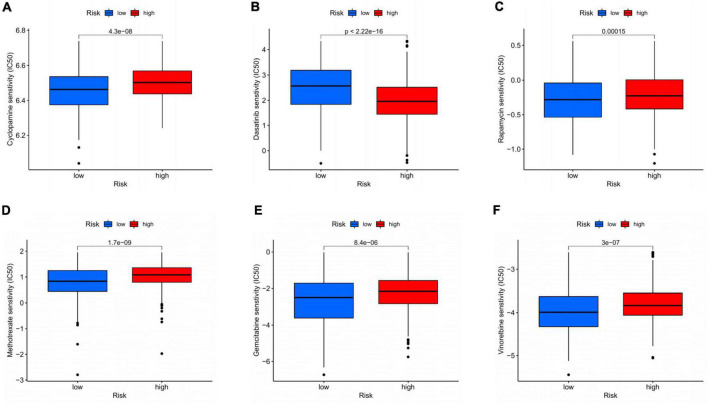
Drug sensitivity analysis based on CDEGs-score. **(A–F)** Relationship between chemotherapeutic drugs and CDEGs-Score. CDEGs, cancer associated fibroblast related differential expression genes.

The sensitivity of patients in the low- and high-risk groups to the chemotherapy drugs selected according to current clinical situation was evaluated. Notably, patients in the high-risk group had lower IC50 value compared to low-risk group with regard to dasatinib. In the low-risk group, IC50 values of cyclopamine, rapamycin, methotrexate, gemcitabine, and vinorelbine were lower relatively (Figures 10A–F). In general, there was indeed remarkable correlation between the CDEGs-Score and drug sensitivity.

## Discussion

Helicobacter pylori (HP) is the predominant species in the human gastric micropopulation, and the gastritis it induces has been established as the greatest single risk factor for GC (22). In clinical studies and animal models, researchers have directly demonstrated that HP eradication can contribute to the prevention of GC in infected individuals without pre-malignant lesions (23, 24). As for pathobiology, the risk for the HP-infected people developing gastric cancer is dependent on multiple factors including HP strain-specific virulence factors, the host genotype, environmental factors such as diet, and the alternations in the immune microenvironment (25–27). Though, the current researches concerning the immune microenvironment mainly focus on the interaction of immune cells and subsequent signaling, such as the NF-κB pathway and inflammasomes, partly neglecting the role of other non-immune cell components with high significance (28). Since several experiments have confirmed HP-related gastric fibroblasts as momentous characters in the genesis and development of HP-related GC, it is meaningful to further discuss their specific mechanism with an overall genome and immune landscape cognition (18, 19). Thus, we sorted the HP-related GC patients into two subtypes with HRGs and thus identified CDEGs, and further analyzed the correlation between their expression and TME as well as the prognosis, finally establishing prognostic models for guiding the personal treatment of HP-related GC patients.

In this study, we screened out 52 HRGs with HP-related GC samples in the GSE84437 dataset. The genetic analysis indicated a high frequency of mutations in HRGs, especially in *TP53* (44%), *RF2* (5%), *PLCG1* (4%), *CASP5* (4%), and *CASP8* (4%). Several human studies have certified the relationship between HP-related GC and the development of *TP53* mutations, the latent mechanism of which may be the selective pressures offered by exogenous exposures for the emergence of mutant TP53 clones (29, 30). The other high-frequency genetic mutations were first reported in HP-related GC providing a novel perspective. More interestingly, we discovered that the expression of most HRGs was upregulated in GC, but had no significant correlation with the CNV alteration, implying their regulation might be controlled by a complex transcriptional regulatory network or epigenetics alternation.

Based on the expression of HRGs, we could divide the GC patients into two subtypes with significant prognosis differences. In subtype B, the patients had better RFS, lower T stage, and higher age. With GSVA enrichment analysis, we discovered that the subtype B was prominently enriched in synthesis and metabolic pathways as well as gene damage repair pathways including mismatch repair, homologous recombination, and base excision repair pathways, which might partly account for its better prognosis. In addition, subtype A was enriched in extracellular matrix remodeling and cell adhesion related pathways, indicating the diversity between the two subtypes in TME. Therefore, we further the relationship between these two subtypes and immune infiltration. Compared with subtype B, subtype A had significantly more activated immune infiltration, including activated B cells, activated CD8 T cells, eosinophils, immature B cells, MDSCs, macrophages, mast cells, regulatory T cells, T follicular helper cells, and type 1 helper cells. Geng et al. constructed a novel immune-related signature for predicting the survival and curative effect of HP-positive GC patients (31). Their model enriched the high-risk group in several immune-related pathways, including B cell receptor signaling pathway, leukocyte transendothelial migration, natural killer cell mediated cytotoxicity, and type 1 and 2 helper cell differentiation. Conversely, some metabolic pathways, such as carbon metabolism, DNA replication, and nitrogen metabolism were upregulated in the low-risk group, which was consistent with our findings. They also verified that the patients with more intense immune infiltration had a worse prognosis.

As the most dominant cell type in the stroma of TME, CAFs can promote tumor progression, metastasis, and angiogenesis with extracellular matrix remodeling (32, 33). Moreover, CAFs also interact with diverse immune infiltration cells regulating the anticancer immunological status of TME (34). The functional enrichment analysis of the DEGs between the two subtypes indicated that these DEGs were highly correlated with extracellular matrix organization, extracellular structure organization, muscle contraction, collagen-containing extracellular matrix, and contractile fiber, which reveal the potential role of CAFs in causing the subtypes’ differences. With EPIC and WGCNA, we discovered a significant synergic relationship between the CAFs and endotheliocytes, affirming the promoting efficacy of CAFs in EMT. The paracrine factors released by CAFs, especially transforming growth factor-β, were regarded as the main approach in modulating EMT (35). More interestingly, we also found that the B cells might have an antagonistic effect on CAFs, offering an unrevealed viewpoint.

Considering the significance of these DEGs and CAFs-related heterogeneity on the clinical outcomes, a CDEGs-Score based on a 21-gene signature identified by Cox regression analysis was constructed for HP-related GC patients. The patients were divided into high- and low-score groups, and the high score was related to poor prognosis. We also further confirmed the efficacy of this novel risk score model with the test, train, and all cohorts. The risk score model had outstanding AUC in predicting OS, and the low risk was significantly associated with better survival. The nanogram based on the risk score and the clinicopathological parameters also showed satisfying performance in predicting the OS of the 1-, 3-, and 5-year. Zhang et al. also developed a prognosis model based on the CAFs subtypes for GC patients, however, our model was aimed at HP-related GC patients achieving more personal and precise prediction (34).

For further analysis of the possible factors determining the survival difference between the two groups, we acquired the distribution variations of the somatic mutations and discovered that the mutations of *MUC16, PCLO, and PIK3CA* were the most essential. Encoding cancer antigen 125 (CA125), *MUC16* is frequently mutated and highly related to the prognosis in various tumors, including cholangiocarcinoma (36–39), ovarian carcinoma, hepatocellular carcinoma, and GC. With two GC cohorts, Li et al. verified that *MUC16*/CA125 mutation was associated with high tumor mutation load and better outcomes in gastric cancer (40), which was consistent with our result. In previous studies, *PLCO* was considered to be correlated with neuropsychosis such as bipolar disorder, major depressive disorder, pontocerebellar hypoplasia type III, etc (41–43). Interestingly, the own definitive molecular features of EBV-positive GC include *PIK3CA* mutations, which may be associated with signaling pathways leading to GC oncogenic process by promoting chronic gastric inflammation (44). Furthermore, these CDEGs were highly related to the immune cells, stromal, and estimate scores, indicating high-risk showed a positive correlation with activated immune infiltration.

Among diverse solid tumors, GC is considered the most common one displaying CAF-mediated chemo-resistance including paracrine, exosomal cargo, extracellular vesicle, and secretomic modes of action (45). Therefore, we further explored the drug sensitivity of frequently used chemotherapeutic agents in GC based on the CDEGs-Score grouping, proving that the low-risk group was more sensitive to cyclopamine, rapamycin, methotrexate, gemcitabine, and vinorelbine. Similarly, Wei et al. demonstrated that CAFs facilitated malignant progression and gemcitabine resistance of pancreatic cancer *via* secreting SDF-1 (46). Our findings also partly accounted for the prognosis difference resulting from the CDEGs classification and provided clinical guidance in treatment. However, the above results were acquired based on the data from public datasets, and more clinical studies were required to verify our novel risk score model for HP-related GC patients.

Nevertheless, our research offered a novel viewpoint for understanding HP-related GC. Based on an overall genome cognition, we screened out HRGs and thus identified two subtypes according to the differential expression. By further enrichment and immune infiltration analysis, we verified the essential role of CAFs in leading this heterogeneity. Eventually, a reliable risk score model based on CDEGs was constructed for predicting the chemotherapeutic efficacy and prognosis. In sum, our systematic study of CAFs and CDEGs will act as a pioneer effort for exploring their roles and value in HP-related GC.

## Conclusion

In summary, we comprehensively analyzed the expression pattern of HRGs and thus identified two subtypes in HP-related GC patients. Based on these two subtypes, the CDEGs-score model was constructed for predicting the prognosis of HP-related GC patients and guiding their precise treatment for the first time. Meanwhile, our study also provides a novel perspective on studying the mechanism for malignant progression in HP-related GC, indicating targeting CAFs may be a potential therapy.

## Data availability statement

The datasets presented in this study can be found in online repositories. The names of the repository/repositories and accession number(s) can be found in the article/Supplementary material.

## Author contributions

RX, LY, ZZ, and YL contributed to the conception and design of the work, the collection and analysis of data, and the writing and editing of the manuscript. YY, DZ, JL, HG, and WX provided editing and writing assistance. All authors contributed to the article and approved the submitted version.

## Footnotes

1
https://www.ncbi.nlm.nih.gov/geo/

2
https://tcga-data.nci.nih.gov/tcga

3
http://www.broad.mit.edu/gsea/msigdb/

4
https://www.r-project.org/
